# Team-Based Learning Among Health Care Professionals: A Systematic Review

**DOI:** 10.7759/cureus.21252

**Published:** 2022-01-14

**Authors:** Tilak Joshi, Pravash Budhathoki, Anurag Adhikari, Ayusha Poudel, Sumit Raut, Dhan B Shrestha

**Affiliations:** 1 Department of Internal Medicine, Mount Sinai Hospital, Chicago, USA; 2 Department of Internal Medicine, Bronxcare Health System, New York, USA; 3 Intensive Care Unit, Nepal Korea Friendship Municipality Hospital, Madhyapur Thimi, NPL; 4 Emergency Medicine, Alka Hospital Pvt. Ltd., Kathmandu, NPL; 5 Internal Medicine, Kathmandu Medical College, Kathmandu, NPL

**Keywords:** medical education, problem-based learning, problem solving, learning, health personnel

## Abstract

Introduced in the 1970s to meet the academic needs of a growing number of students with relatively stagnant faculty, team-based learning (TBL) has revolutionized the modern classroom structure. Contrary to the traditional didactic model where the teacher assumes the central role and students are passive listeners, TBL participants are actively involved in the learning process. Teachers act as facilitators while the TBL participants work in groups to solve problems through engagement with their peers. The objective of the article is to conduct a systematic review on team-based learning using the Preferred Reporting Items for Systematic Reviews and Meta-Analyses (PRISMA) guideline.

The studies were searched in databases like PubMed®, Scopus®, Embase®, and PubMed Central® using appropriate keywords. Two authors screened the papers, and a third author resolved the conflicts. This was followed by a bibliographic review based on the references of the selected study and bias assessment using the Joanna Briggs Institute (JBI) critical appraisal tool.

The team-based learning model is increasingly being used by different institutions globally. TBL and traditional lecture-based teaching outcomes revealed that TBL participants performed better in academic, clinical, and communication domains. In addition, TBL enhanced learners' engagement, collaborative spirit, and satisfaction. Our study results are similar to the prior meta-analysis and systematic review. Nevertheless, this systematic review remains more comprehensive, up-to-date, and inclusive thus far.

Team-based learning is a pragmatic and superior approach to learning among health care professionals. It has resulted in better academic, clinical, and communication outcomes. This finding spans all the medical and allied professions studied in this systematic review.

## Introduction and background

In education, developing and strengthening the skills such as problem-solving, critical thinking, interpersonal communication skills are crucial. Therefore, it is necessary to create an educational environment to link theoretical training with real-life situations [1,2]. Traditionally, lecture-based teaching was the most common way of disseminating information. A class of students facilitated by a single teacher was the universal method of teaching [1]. Lecture-based learning is widely the mainstream learning method globally due to the constraints of teaching resources. However, it was deemed to be tutor-centered. The learners described it as passive and less engaging. Therefore, in recent decades, problem-based learning (PBL) and team-based learning (TBL) methods are gaining popularity as more engaging and productive learning modalities to improve the theoretical knowledge into practice. Problem-based learning is an instructional method that emphasizes learner-led, small group learning. Learners benefit from working in facilitated groups to solve complex, unstructured problems that simulate "real-world" scenarios [3]. The sum of all these elements makes the teaching-learning activity uniquely motivating and intellectually stimulating [4]. In the didactic lecture method, learners are relatively passive in the knowledge acquisition process, whereas the team-based and problem-based learning pedagogy models demand active involvement and engagement.

Team-based learning (TBL) started in 1970, which saw a dramatic increase in the number of medical students in medical school. However, the number of faculty to teach the students was relatively static. A logistical challenge arose to incorporate a large number of students into problem-based learning. During this period, educator Larry Michaelson came up with an idea to divide students into teams with less than ten students initially in his business school. The classroom teaching activity would be based on the "4S" framework which he had devised. Students would work "on a *significant *problem, the *same *problem, where they had to make a *specific *choice and make a *simultaneous *report" [5]. TBL comprises pre-class preparation, individual readiness assurance test (IRAT), team readiness assurance test (TRAT), followed by feedback and problem-solving activities [6]. The benefit of this method was that the students were deeply engaged with the content and knew how to apply the same. This ushered a new beginning of team-based learning in health care education [5].

Team-based learning is gaining popularity all around the world as a form of active learning [7,8]. It enhances learning motivation and encourages students to apply knowledge-based materials in problem-solving and integrate them into practice. As a result, medical schools from various countries, including the USA, China, Japan, Korea, India, Singapore, Oman, and Australia, have adopted team-based learning [9]. In light of the shift towards team-based learning, we conducted this review to evaluate the impact of team-based learning among health professionals such as practicing physicians, resident physicians, medical students, nursing, pharmacy, and dentistry students in different countries.

## Review

Methods

Our systematic review was based on the Preferred Reporting Items for Systematic Reviews and Meta-Analyses (PRISMA) guideline [10].

Database Search and Screening

We searched PubMed®, Scopus®, Embase®, and PubMed Central® till February 17, 2021, to identify the studies using Medical Search Heading (MeSH) and keywords containing "team learning," "collaborative learning," "cooperative knowledge," "health care workers," "health care professionals" and "medicine." Electronic search details are available in Appendix 1. Two independent reviewers did the screening, and a third reviewer resolved the conflict between the two reviewers using Covidence software. A bibliographic review was conducted by meticulous analysis of the references listed in the selected articles.

Selection of Studies

The inclusion criteria for study selection were original articles with quantitative tools for measuring the impact of team-based learning, emphasizing randomized controlled trials followed by cohort, case-control, and cross-sectional studies. The interest of study spans all medical professions. However, for brevity, only the pertinent quantitative outcomes were analyzed.

Data Extraction

Three authors carried out data extraction, and a consensus was achieved via a virtual meeting held as required during the study period. An extraction template was created, and each author followed the template during article extraction. A single author extracted a single study data to avoid conflicts which two other authors then cross-checked.

Data Synthesis

 A systematic review of extracted articles was done. Studies with similar outcome measures were grouped and analyzed. Studies or sections of studies with analysis of participants' perceptions about a particular study model were excluded from being subjective. The characteristics of the detailed studies are analyzed and tabulated. Frequency and percentages were used to describe the baseline characteristics of the involved participants and outcomes. Means and standard deviations were used to represent the study outcomes. Meta-analysis was not possible due to heterogeneity in the designs and outcome measures of the different studies.

Assessment of Bias

We used the Joanna Briggs Institute (JBI) critical appraisal tool for the assessment of bias of the included studies (see Tables 1-4) [11]. 

**Table 1:** JBI critical appraisal for randomized controlled trials JBI - Joanna Briggs Institute; RCT - randomized controlled trials

Questions (Yes, No, Unclear, Not applicable)	Carrick et al. [12]	Huang et al. [13]	Yan et al. [14]	Zeng et al. [15]	Das et al. [16]	Athanassaki et al. [17]	Zingone et al. [18]	Liaw et al. [19]	Riddell et al. [20]
Was proper randomization used for the assignment of participants to treatment groups?	Yes	Yes	Yes	Yes	Yes	Yes	Yes	Unclear	Yes
Was allocation to treatment groups concealed?	No	No	No	No	No	No	No	No	No
Were treatment groups similar at the baseline?	Yes	Yes	Yes	Yes	Yes	Yes	No	Yes	Unclear
Were participants blind to treatment assignment?	No	No	No	No	No	No	No	No	No
Were those delivering treatment blind to treatment assignment?	No	No	No	No	No	No	No	No	Yes
Were outcomes assessors blind to treatment assignment?	No	No	No	No	No	No	No	Unclear	Unclear
Were treatment groups treated identically other than the intervention of interest?	Yes	Yes	Yes	Yes	Yes	Yes	Yes	Unclear	Yes
Was follow-up complete and if not, were differences between groups in terms of their follow-up adequately described and analyzed?	Yes	Yes	Yes	Yes	Yes	Yes	Yes	Unclear	No
Were participants analyzed in the groups to which they were randomized?	Yes	Yes	Yes	Yes	Yes	Yes	Yes	Yes	Yes
Were outcomes measured in the same way for treatment groups?	Yes	Yes	Yes	Yes	Yes	Yes	Yes	Yes	Yes
Were outcomes measured reliably?	Yes	Yes	Yes	Yes	Yes	Yes	Yes	Yes	Yes
Was appropriate statistical analysis used?	Yes	Yes	Yes	Yes	Yes	Yes	Yes	Yes	Yes
Was the trial design appropriate, and any deviations from the standard RCT design (individual randomization, parallel groups) accounted for in the conduct and analysis of the trial?	Yes	No	Yes	Yes	Yes	Yes	Yes	No	No
Critical appraisal	Include	Include	Include	Include	Include	Include	Include	Include	Include

**Table 2:** JBI critical appraisal for non-randomized experimental studies JBI - Joanna Briggs Institute

Questions (Yes, No, Unclear, Not applicable)	Is it clear in the study what the 'cause' and the 'effect' (i.e., there is no confusion about which variable comes first)?	Were the participants included in any comparisons similar?	Were the participants included in any comparisons receiving similar treatment/care, other than the exposure or intervention of interest?	Was there a control group?	Were there multiple measurements of the outcome, both pre and post the intervention/exposure?	Was follow-up complete and if not, were differences between groups in terms of their follow-up adequately described and analyzed?	Were the outcomes of participants included in any comparisons measured in the same way?	Were outcomes measured reliably?	Was appropriate statistical analysis used?	Critical appraisal
Badiyepeymaie Jahromi et al. [21]	Yes	Yes	No	No	No	Yes	Yes	Yes	Yes	Include
Faezi et al. [22]	Yes	Yes	No	Yes	Yes	Yes	Yes	Yes	Yes	Include
Boyson-Osborn et al. [23]	Yes	Yes	No	Yes	Yes	No	Yes	Yes	Yes	Include
Ghorbani et al. [24]	Yes	Yes	No	Yes	Yes	Yes	Yes	Yes	Yes	Include
Halasa et al. [25]	Yes	Yes	No	Yes	Yes	Yes	Yes	Yes	Yes	Include
Hemmati Maslakpak et al. [26]	Yes	No	No	Yes	Yes	Yes	Yes	Yes	Yes	Include
Jafari et al. [27]	Yes	Yes	No	Yes	Yes	Yes	Yes	Yes	Yes	Include
Jafarkhani et al. [28]	Yes	No	No	Yes	Yes	Yes	Yes	Yes	Yes	Include
Jost et al. [29]	Yes	Yes	No	Yes	No	Yes	Yes	Yes	Yes	Include
Wiener et al. [7]	Yes	No	No	Yes	No	Yes	Yes	Yes	Yes	Include
Tahir et al. [30]	Yes	Yes	No	No	No	Yes	Yes	Yes	Yes	Include
Brandler et al. [31]	Yes	Yes	No	No	Yes	Yes	Yes	Yes	Yes	Include
Vazquez-Garcia et al. [32]	Yes	Yes	No	No	No	Yes	Yes	Yes	Yes	Include
Chandelkar et al. [33]	Yes	Yes	No	No	Yes	Yes	Yes	Yes	Yes	Include
Berg et al. [34]	Yes	Yes	No	Yes	No	Yes	Yes	Yes	Yes	Include
Rezaee et al. [2]	Yes	Yes	No	No	No	Yes	Yes	Yes	Yes	Include
Burgess et al. [35]	Yes	Yes	Yes	No	No	Unclear	Yes	Yes	Yes	Include
Cevik et al. [36]	Yes	No	Yes	No	No	Unclear	Yes	Yes	Yes	Include
Milzman et al. [37]	Yes	Unclear	Unclear	No	No	Unclear	Unclear	Yes	Yes	Include
Tan et al. [38]	Yes	Yes	No	Yes	No	Yes	Yes	Yes	Yes	Include

**Table 3:** JBI critical appraisal for cohort study and retrospective cohort JBI - Joanna Briggs Institute

Questions (Yes, No, Unclear, Not applicable)	McMullen et al. [39]	Lein et al. [40]	Saudek et al. [41]	Levine et al. [42]
1. Were the two groups similar and recruited from the same population?	Yes	Yes	Yes	Yes
2. Were the exposures measured similarly to assign people to both exposed and unexposed groups?	Yes	Yes	Yes	Yes
3. Was the exposure measured validly and reliably?	Yes	Yes	Yes	Yes
4. Were confounding factors identified?	No	No	No	No
5. Were strategies to deal with confounding factors stated?	No	No	No	No
6. Were the groups/participants free of the outcome at the start of the study (or at the moment of exposure)?	No	No	No	No
7. Were the outcomes measured validly and reliably?	Yes	Yes	Yes	Yes
8. Was the follow-up time reported and sufficient to be long enough for outcomes to occur?	Yes	Yes	Yes	Yes
9. Was follow-up complete, and if not, were the loss reasons to follow up described and explored?	Yes	Yes	Yes	No
10. Were strategies to address incomplete follow-up utilized?	No	No	No	No
11. Was appropriate statistical analysis used?	Yes	Yes	Yes	Yes
Overall appraisal	Include	Include	Include	Include

**Table 4:** JBI critical appraisal for cross-sectional studies JBI - Joanna Briggs Institute

Questions (Yes, No, Unclear, Not applicable)	Ihm et al. [43]	Balwan et al. [44]	Kelly et al. [45]
1. Were the criteria for inclusion in the sample clearly defined?	Yes	Yes	Unclear
2. Were the study subjects and the setting described in detail?	Yes	Yes	Yes
3. Was the exposure measured validly and reliably?	Yes	Yes	Yes
4. Were objective, standard criteria used for measurement of the condition?	Yes	No	Yes
5. Were confounding factors identified?	Unclear	Unclear	No
6. Were strategies to deal with confounding factors stated?	Unclear	Unclear	No
7. Were the outcomes measured validly and reliably?	Yes	Yes	Yes
8. Was appropriate statistical analysis used?	Yes	Yes	Yes
Overall Appraisal	Include	Include	Include

Results

**Figure 1:**
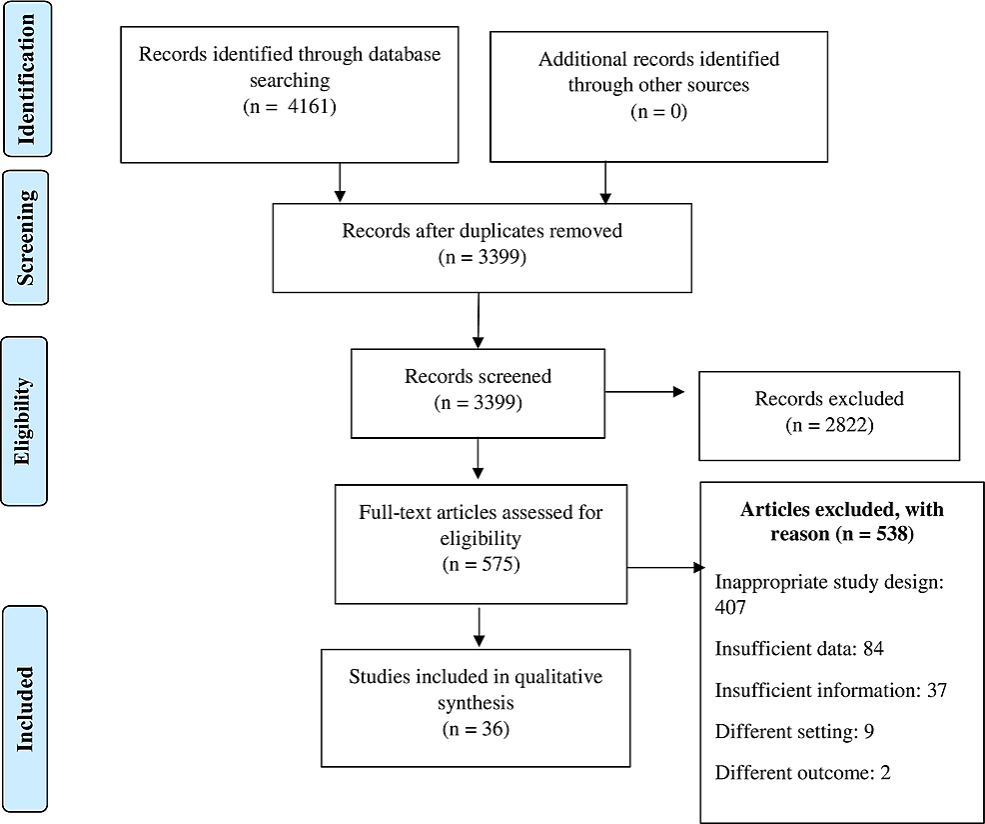
PRISMA flow diagram PRISMA - Preferred Reporting Items for Systematic Reviews and Meta-Analyses

We identified a total of 4161 studies after thorough database searching. After the removal of duplicates, we screened the title and abstract of 3399 studies. A total of 2795 studies were excluded, and we assessed the full-text of 603 studies, excluding 538 for definite reasons. Thus, we included 36 studies in our final qualitative analysis. The following is summarized in the PRISMA flow diagram (Figure 1). Among included 36 studies, ten were from the USA, seven were from Iran, three were from China, two each from India and the United Kingdom, and the rest were from other countries (Table 5). Twenty-eight studies were carried on medicine faculty, six among nurses and two among physicians. Of those studies, 20 were non-randomized experimental studies, nine were randomized controlled trials (RCTs), then four cohorts, and three were cross-sectional studies.

**Table 5:** Narrative summary of the included studies TBL - team-based learning; TL - traditional learning; IRAT - Individual Readiness Assurance Test; TRAT - Team Readiness Assurance Test; MCQ - multiple choice question

#	Study	Country	Experimental group (TBL)	Control group	Limitations
1.	Badiyepeymaie Jahromi et al. 2016 [21]	Iran	Mean score of final exam: Mean +/- SD		No control group
			Web quest (N=38): 67.08+/-6.43		
			TBL (N=39): 59.08+/-6.43		
2.	Balwan et al. 2015 [44]	USA	1) Survey: both resident and faculty agreed that TBL should be included in the future sessions		Lack of generalizability
			2) Average score of Group Readiness Assurance Test (GRAT) was increased by 22% from Individual Readiness Assurance Test (IRAT)		
3.	Faezi et al. 2018 [22]	Iran	Classroom engagement survey (CES): Mean +/- SD	Classroom engagement survey (CES): Mean +/- SD	Quasi-experimental study
			Team-based learning (TBL): 26.7+/-3.70	Lecture-based (LB): 23.80+/-4.35	Short period of TBL sessions
4.	Boysen-Osborn et al. 2016 [23]	USA	Correct percentage:	Correct percentage:	Students skipped the podcast sessions
			a) combined test score fllipped classroom/team-based learning: (FC/TBL); (N=95): 95.1%	a) combined test score LB (N=259): 93.5%	
			b) 7 case fill in the blank test FC/TBL: 95.1%	b) 7 case fill in the blank test LB: 94.1%	
			c) 50 Multiple choice question (MCQ) score FC/TBL: 90%	c) 50 MCQ score LB: 88%	
5.	Carrick et al. 2017 [12]	UK	1) Live classroom scores: Mean +/- SD; Post-test: 86.1 +/-5	1) Live classroom scores: Mean +/- SD; Pre-test: 46.9+/-9.8	Technical issues
			2) Online classroom scores: Mean +/- SD; Post-test: 86 +/-5.3	2) Online classroom scores: Mean +/- SD; Pre-test: 48.3 +/-10.4	Cost for the training limited the resources
6.	Cevik et al. 2019 [36]	UAE	1) Score percentage - the same topic learned during 2nd year using TBL: 71.4%	1) Score percentage - topic learned during 1st year using didactic and case discussion: 63.9%	Case discussion did not entirely match the traditional didactic learning method
			2) The topic was studied in the second year by didactics and case discussion (75.5%)	2) The topic was studied in the first year by didactics (70.3%)	
			3) Second year TBL (70.0%)	3) Second-year didactic study of the same topic (75.2%)	
7.	Ghorbani et al. 2014 [24]	Iran	1) Final examination scores (max score of 8) TBL: 6.5	1) Final examination scores (max score of 8) Lecture: 6	Students were on a course for a long time
			2) Pre-test vs post-test score (max score of 5) TBL: 1.5 vs 2.6	2) Pre-test vs post-test score (max score of 5) Lecture: 1.5 vs 2.2	Methodology relied upon the instructor
8.	Halasa et al. 2020 [25]	Jordon	Academic performance (mean score from all three examinations)	Academic performance(mean score from all three examinations)	Small sample size
			The experimental group (N=59): 77.77	Control group (N=66): 72.33	
9.	Hemmati Maslakpak et al. 2015 [26]	Iran	Intervention group score (max score of 40)	Control group score (max score of 40)	Small sample size
			Pre-test: 13.39	Pre-test: 15.15	
			Post-test: 31.07	Post-test: 17.22	
10.	Huang et al. 2016 [13]	China	Student's performance of TBL on Opthalmology exam: Mean +/- SD		Small sample size
			IRAT: 63.78+/-9.30		
			GRAT: 75.65+/-7.40		
			Group application problem (GAP): 4.247+/-0.45		
			Final examination scores (FES): 76.77+/-4.16		
11.	Jafari et al. 2014 [27]	Iran	Score comparison based on gender (out of 20; Male [M]: Female [F])		Different topics for different methods
			Lecture (M:F= 11.52 : 12.19)		
			IRAT (M:F = 13.36 : 15.24)		
			GRAT (M:F = 14.91 : 16.74)		
			Final (M:F = 13.13 : 14.46)		
12.	Jafarkhani et al. 2020 [28]	Iran	1) Cooperative flipped group (mean)	1) control group (mean)	Small sample size
			Pre-test: 3.56	Pre-test: 3.24	
			Post-test: 15.71	Post-test: 12.75	Lack of gender diversity
			2) Individual flipped group (mean)		
			Pre-test: 4.10		
			Post-test: 13.38		
13.	Jost et al. 2017 [29]	Germany	Key feature problem examination showed better results with TBL (N=17) in comparison to non-TBL (N=15)		Different topics for another method of learning
14.	McMullen et al. 2013 [39]	UK	Class engagement survey (SEC) score: score range from 5 to 40	Class engagement survey (SEC) score: score range from 5 to 40	Lack of generalizability
			TBL: 32.3	TL: 25.5	
15.	Lein et al. 2017 [40]	Korea	Grade point average (undergraduate): Mean +/- SD	Grade point average (undergraduate): Mean +/- SD	Nonrandomization
			Basic skills TBL: 3.64+/-0.23	Basic skills traditional class: 3.59+/-0.27	Lack of control group
			Cardiopulmonary TBL: 3.65+/-0.23	Cardiopulmonary Traditional: 3.60+/-0.27	
16.	Wiener et al. 2009 [7]	Austria	1) Passed percentage of the students TBL: 31.1%	1) Passed percentage of the students non-TBL: 17.2%	Non-randomization
			2) Outcome of the final exam (exam block 4; a maximum score of 50 points with a passing threshold of 30) TBL: 28+/-9 (Mean +/- SD)	2) Outcome of the final exam (exam block 4; a maximum score of 50 points with a passing threshold of 30) non-TBL:22+/-9 (Mean +/- SD)	
17.	Tahir et al. 2020 [30]	Saudi Arabia	1) Flip The Classroom (FTC): Mean +/- SD	1) Traditional Lecture (TL): Mean +/- SD	Lack of generalizability
			Overall score: 47.3+/-6.1	Overall score: 42.7+/-5.9	
			Multiple-choice questions (MCQs): 13.4+/-2.7	MCQs: 12.3+/-2.4	
			Objective Structured Clinical Examination (OSCE): 33.9+/-4.3 vs 30.4+/-4.7	OSCE: 30.4+/-4.7	
18.	Tan et al. 2011 [38]	Singapore	Mean percentage change in score from baseline pre-test	Mean percentage change in score from baseline pre-test	Small sample size
			TBL: 8.8% (post-test 1 - i mediately)	PL (passive learning): 4.3 (post-test 1 - immediately);	Modified TBL due to lack of time
			TBL: 11.4% (post-test 2- after 48 hours); p=0.001	PL: 3.4 (post-test 2 - after 48 hours)	Similarity between post-test 1 and 2
19.	Riddell et al. 2017 [20]	USA	Mean	Mean	Use of single lecture topic
			1) Low back pain: flipped (N=38)	1) Low back pain: lecture(N=37)	
			Pre-test: 0.66	Pre-test: 0.63	
			Post-test: 0.77	Post-test: 0.76	
			Retention test: 0.70	Retention test: 0.75	
			2) Headache: flipped (N=37)	2) Headache: lecture (N=36)	
			Pre-test: 0.78	Pre-test: 0.82	
			Post-test: 0.80	Post-test: 0.75	
			Retention test: 0.84	Retention test: 0.81	
20.	Saudek et al. 2015 [41]	USA	Pre Blood Disorders (BD) module: Mean +/- SD	Post BD module: Mean +/- SD	Historical controls
			Institutional score: 0.65+/-0.19	Institutional score: 0.70+/-0.21	
			National score : 0.62 +/-0.15	National score: 0.64+/-0.15	
21.	Yan et al. 2018 [14]	China	Average score (out of 100): Mean +/- SD	Average score (out of 100): Mean +/- SD	Small sample size
			TBL: 81.70 +/-8.53	TL: 74.4 +/-8.27	Exchange of opinions between the participants during the study
22.	Zeng et al. 2017 [15]	China	1) Individual terminal test I (Mean +/- SD) TBL: 19.85+/-4.20	1) Individual terminal test I (Mean +/- SD) Lecture-based learning (LBL): 19.70 +/-4.61	Small sample size
			2) Individual terminal test II (Mean +/- SD) TBL: 19.15+/-3.93	2) Individual terminal test II (Mean +/- SD) LBL: 17.46 +/-4.65	
23.	Ihm et al. 2019 [43]	Korea	1) Correct answer rate		Lack of generalizability
			IRAT: Higher GPA > Lower GPA		
			TRAT: Higher GPA > Lower GPA		
			Final exam: Higher GPA>Lower GPA		
			2) Correct answer rate in the higher and lower group(both revealed similar findings)		
			IRAT: Factual knowledge > Clinical reasoning		
			TRAT: Factual knowledge > Clinical reasoning		
			Final exam: Clinical reasoning > Factual knowledge		
24.	Das et al. 2019 [16]	India	1) Score in test assessing problem solving skills (total marks = 20); Mean +/- SD	Traditional lecture (N- 46) : Mean +/- SD	Pilot study
			TBL (N=48) : 8.8+/-3.7	8.8+/-2.7	
			2) Score in test assessing problem solving skills (total marks = 20); N=16; Mean +/- SD		
			High achievers : 11.25 +/-3.2 (TBL)	9.3 +/-2.3 (TL)	
			Low achievers : 6.2+/-2.5 (TBL)	7.8+/-3.8 (TL)	
25.	Brandler et al. 2014 [31]	USA	1) IRAT and GRAT were compared first through fourth TBL sessions: results were variable		Small sample size
			2) Peer evaluation tool: the quality of team learning was scaled 0(none of the time) to 6 (all of the time) The team performance survey received mean scores ranging from 5.3 ± 0.9 to 6.0 ± 0.0		
26.	Vázquez-García et al. 2018 [32]	Mexico	Average score in CP (collaborative phase was found to be 70% greater than IP (individual phase): Mean +/- SD		Small sample size
			Average subtopic quizzes score CP vs IP (69.8 +/- 2.7 vs. 47.2 +/- 2.2)		
			Average global assessment quizzes score CP vs IP (61.0 +/- 0.6 vs. 44.8 +/- 0.8)		
27.	Chandelkar et al. 2014 [33]	India	MCQ test of 40 marks, mean percentage score of tests are:		Small group students were large in number
			Test I: 27.83		
			Test II: 50.66		
			Test III: 78.66		
			Feedbacks after small group teaching: A good percentage of people thought it helped answer the MCQ test, improved learning, and showed interest in similar exercises in the future.		
28.	Berg et al. 2012 [34]	Denmark	Test score results from high to low score: students doing individual quizzes > students doing group quizzes > controls		Limited time for group discussion
29.	Burgess et al. 2016 [35]	Australia	IRAT: Score increase from the Week 1 assessment (median = 2) to the Week 2 assessment (median = 3.5), with a median difference in score of 1.5. (n = 18)	Not applicable	Small sample size; only two TBL iterations
			Participants number and score improvement between weeks:		
			12 participants: 1 to 6 points		
			4 participants: no improvement		
			3 participants: improved by 2 points		
			1 participant: improved by 3 points		
			2 participants scored lower in Week 2		
			TRAT: all teams (except one) scored lower on week 2		
			Team 1: 67.5% to 72.5%		
			Team 2/3: 80% to 70%		
			Team 4: 75% to 73%		
30.	Zingone et al. 2010 [18]	USA	Mean scores (Mean +/- SD) : 3.7 ±6 0.2	Mean scores (Mean +/- SD) 3.3 ±6 0.5	Limited sample size
31.	Athanassaki et al. 2020 [17]	USA	Team Readiness Assessment Test/ Team Application Problems (tRAT/tAPP) (Mean=94%; range: 83% to 100%)	Individual Readiness Assessment Test/ Individual Application Problems (iRAT/iAPP) (Mean=76% range: 60% to 89%)	Trust placed on the fellows to not use the outside resources
					Few questions were straightforward; objectives were longer compared to other studies
32.	Liaw et al. 2020 [19]	Singapore	Overall communication performance post-test scores:	Overall communication performance post-test scores:	Immediate post-test on team performance
			Virtual (Mean+/- SD) 22.60±5.31	Live simulation group (Mean +/- SD): 23.97±4.55	Single-center study
33.	Rezaee 2015 [2]	Iran	N=40; Mean +/- SD	N=41; Mean +/- SD	Small sample size
			Pre: self-regulation 58.72±5.02; the desire for learning 55.26±5.11; self-management 46.6±4.37; total 68.47±6.41	Traditional (n=41) 13.24 ±2.01	Acceptance of traditional method as a comparator group
			Post: self-regulation 59.06±4.89; the desire for learning 55.44±4.61; self-management 50.6± 4.46; total 69.90 ±5.36		
34.	Levine et al. 2004 [42]	USA	Revised curriculum (Mean +/- SD): M=72.9± 8.32, N=133	Lectures only (Mean +/- SD) Class of 2003: M=70.3±8.18, N=147	Controls from the end of the previous academic year may have had a different clinical experience which may impact engagement
				Class of 2004: M=69.6±9.35, N=130	The National Board of Medical Examiners (NBME) test scores may be influenced by multiple factors
			Overall engagement score, (d=1.13) for the team learning activities (M=4.24, SD=0.61, N=281) compared to the replaced lectures	Overall engagement score: M=3.46, SD=0.95, N=71	
35.	Kelly et al. 2005 [3]	USA	Team learning:	Lecture	
			Engaged with each other: 51%	Engaged with each other: 9%	
			Engaged with teacher: 21%	Engaged with teacher: 58%	
			Self-engaged (reading/writing/ not visibly interacting with others ): 28%	Self-engaged (reading/script/ not visibly interacting with others): 33%	
36.	Milzman et al. 2013 [37]	USA	Critical action (8) in Intensive Care Unit (ICU) resuscitation scenario:	Critical action (8) in ICU resuscitation scenario:	A pilot project
			Mean scores: 6.5 actions in a mean	Mean scores: medical: 4.3±3.4, nursing: 3.5 ±3.1	
			Meantime to completion: 19.4min	Meantime to completion: medical: 24.8 mins, nursing =25.2 mins	

A survey among internal medicine residents and faculties with a standard 4+1 block supplemented with TBL was performed at Hofstra North Shore-LIJ. Residents were divided into five cohorts, where each cohort rotated into ambulatory clinics in their every fifth week. Both residents and faculty agreed that TBL should be included in future sessions. Also, the group readiness assurance test scores (GRAT) increased by 22% from the individual readiness assurance test (IRAT) [44].

Another study in the United States was done among medical students to analyze the effectiveness of TBL over classroom teaching for advanced cardiac life support. Flipped class/team-based learning (FC/TBL) advanced cardiac life support (ACLS) course in 2015 (N=95) lasted 27.5 hours (10.5 hours TBL, nine hours podcast, and eight hours of a small-group simulation) whereas lecture-based ACLS course in 2012-2014 (N=259) lasted 20 hours (12 hours of lecture and eight hours of a small-group simulation). Students were assessed with 50 multiple choice questions (MCQ), seven fill-in clinical cases, and 20 cardiac rhythm tests. Students who attended FC/TBL ACLS courses scored more in MCQ and clinical cases than students attending the lecture-based ACLS course. Also, more students failed one of the three tests in the lecture-based approach. All findings were statistically significant. All data were compared using the Kruskal-Wallis rank-sum test [23].

Badiyepeymaie Jahromi et al. conducted a study in Iran among nursing students to compare the effect of Webquest and team-based learning (TBL) on students' self-regulation and academic achievement. A total of 77 nurses were divided into two groups and were introduced with Webquest or a team-based learning approach to learn psychiatry curriculum. The final score out of 100 showed 67.08+/-6.43 in Webquest and 59.08+/-6.43 in team-based approach learning with a p-value of 0.002. Guglielmino's self-directed learning readiness scale (SDLRS; a 41 item questionnaire), having three self-management, learning engagement, and self-control sections, was measured individually out of 100. Data showed Webquest 18.35+/-3.14 and TBL 21.94+/-12.50, but differences were statistically insignificant. Buford's self-regulation questionnaire (14 items) was also compared between the two groups but was statistically negligible as well [21]. 

Another study in Iran analyzed the effectiveness of TBL in a rheumatology course. Out of 84 participants, 34.88% were males, and 65.11 % were females. The mean age of the students was 22+/-2.0 years. Faezi et al., however, conducted a quasi-experimental study to compare team-based learning with conventional lecture-based education. The classroom engagement survey (CES) was performed with a reference score of 24. TBL showed increased engagement in the classroom with a score of 26.7+/-3.70 (p=0.0001), whereas a lecture-based learning score of 23.80+/-4.35 was statistically insignificant (p=0.09). IRAT and TRAT were 80.53 and 10.25 respectively out of 11 with a p-value of 0.001. Both groups' mean exam scores showed a decreasing trend when moving from the first assessment to the third. But the effect of the type of learning approach and time for evaluation on those scores were statistically insignificant [22].

In the United Kingdom, a randomized controlled trial was undertaken to test the efficacy of online learning and traditional learning compared to flipped classroom learning. The participants included scholars with active health care practice who desired to study the neuro-otology curriculum. Total participants (N=274) were randomly divided into two groups: an online learning group (using adobe connect) and traditional classroom learning, and the number of females in each group were distributed equally. The mean age of participants was 38.5 years. Pre-test and post-test scores were compared with the two-sample paired test between the two groups, but there was no significant difference in scores with a p-value of0.9195**. **The following interesting finding includes decreased scores of live classroom males compared to females, whereas this finding was not evident in an online classroom [12].

A study in the United Arab Emirates (UAE) was done to analyze the effectiveness of TBL in comparison to didactic lectures in terms of knowledge gain and students' perceptions. Final-year medical students attending the emergency medicine (EM) clerkship from two successive years were included. In the first year of EM clerkship, topics were taught by didactic presentation and case discussions. In contrast, in the second year of EM clerkship, eight topics were provided through TBL, and three topics were provided through didactic presentations and case discussion-based learning. Subject learned during the first year using didactic lectures and case discussions showed a mean score of 63.9; however, the same topic learned during the second year using TBL had a mean score of 71.4% (p<0.001). ANOVA-RM was used for the analysis. The average response of the participants towards the survey using the Likert scale was positive(≥4 out of 5) for all factors such as level of engagement, understanding, consistent attention, and learning outcome [36].

The effectiveness of TBL was also assessed in physical therapy students at the Shiraz University of Medical Science, Iran. Thirty students underwent a lecture-based (LB) learning approach and a team-based learning approach. The final exam score was better with TBL (6.5 vs. 6; p<0.01). Comparison with the paired sample t-test of pre-test and post-test scores revealed improved post-test score with TBL compared to LB learning (p<0.01). A survey to gauge the satisfaction of TBL using a 5-point Likert scale showed an average difference of 0.5 points where the participants pointed that TBL was better in terms of understanding the anatomical concepts and encouraging problem-solving skills, group discussions, and interactions [24].

A survey was done among Jordan's second-year nursing students (N=125) to study the effectiveness of blended and flipped learning compared to traditional learning. Students were divided into experimental (blended with flipped learning) and control groups (traditional learning without flipped classrooms). Characteristics of the experimental group were: N=59; M:F=13/46; average age 19.6 in male and 19 in female and for the control group were: N=66; M:F=13/53; average age 19.8 in male and 18.8 in female). This study demonstrated that the academic performance in the examinations showed statistically significant increased scores with an experimental group (77.77 vs. 72.23) [25].

One of the quasi-experimental studies gauging the effectiveness of team-based learning in third-grade nursing students in learning nervous system examination with fifth-semester students in the intervention group and sixth-semester students in the control group was conducted by Hemmati Maslakpak et al.. Pre- and post-test scores of the intervention and the control group analyzed by paired t-test were 13.39 vs. 31.07 (p<0.001) and 15.15 vs. 17.22 (p<0.145), respectively. In the team-based learning, group means score in nursing students in GRAT was higher than IRAT [26].

At Sun Yat-sen University, 99 medical students volunteered in a study to analyze the effectiveness of team-based learning in ophthalmology clerkship. This study compared the traditional lecture module with the TBL module [13]. The performance of students on the TBL module showed score on GRAT was greater than IRAT without any statistical significance after analysis with paired t-test. Participants strongly agreed that TBL helped them learn, influencing their learning process and attitude, promoting cooperative learning, and highly facilitating the learning process. TBL session was helpful to learning for 57.65% of participants [13].

Another study in Iran was conducted to analyze the effectiveness of team-based learning compared to the traditional learning method regarding student learning. Participants were undergraduate students at the school of rehabilitation, with 32 males and 38 females. Neurology courses were divided into two halves: the first half receiving the lecture-based method and the second half receiving the TBL method. Scores were compared based on gender, and the scores for lecture-based method, IRAT, GRAT, and final exam were M:F=11.52:12.19, p<0.068; M:F = 13.36:15.24, p<0.001; M:F=14.91:16.74, p<0.001; M:F=13.13:14.46, p<0.001 respectively. It showed improvement in scores after the application of a team-based learning approach. Mean differences were measured using the two-sample t-test. Also, increased satisfaction of TBL compared to the lecture method was evident in 81.3% of the participants [27].

In another study, 20 male and 41 female medical students were randomly divided into cooperative flipped, individual flipped, and control groups. In both experimental groups, they watched videos, read study materials, and worked on questions and exercises before attending the class. In cooperative flipped, three groups were formed with students with low, mid, and high scores. ANOVA-test was used to analyze the pre-test and post-test scores, which revealed that the cooperative flipped group had a better response in post-test than with an individual scanned group [28].

A pilot study was done in Germany on students joining neurology courses in 2012/13 to determine the usefulness of TBL on clinical reasoning skills. Examination showed better results with TBL in comparison to non-TBL (p=0.026). However, better results were not seen in the TBL group in multiple-choice question examination, questions referring to topics of seminar/TBL and questions not referring to topics of seminar/TBL with a p-value of 0.303, 0.473, 0.518, respectively [29].

At the most extensive psychiatry program, psychiatry residents in the UK were divided into groups using a line-up method based on prior knowledge in addiction psychiatry by McMullen et al.. There was an equal number of males and females in the study. Group completed the TBL module, which was co-facilitated by a researcher in TBL training. The class engagement survey (score from 5 to 40) showed a positive response with TBL in comparison to traditional learning (32.3 vs. 25.5; p<0.001). The feedback questionnaire also revealed a positive response with TBL except for easiness to complete the pre-session reading and feeling of preparedness for the IRAT [39].

A Korean study was done to analyze the effectiveness of team-based learning in academic outcomes in an entry-level doctor of physical therapy. Traditional learning groups and TBL were compared for basic skills and cardiopulmonary knowledge. It was a continuous study, so the number of participants varied yearly from 31 to 50. The result showed a slight improvement with TBL but was not statistically significant [40].

A study in Austria was conducted to determine the impact of team-based learning on the education of first-year medical students. The total participants were 386, out of which 55% were females. TBL method stood superior to the traditional learning method by showing increased final scores and pass percentage. Data were also stratified based on gender, showing a statistically significant large increase in final scores in males compared to females [7].

A Saudi Arabian study was performed taking female final year medical students to study the effectiveness of learning obstetrics and gynecology in a flipped classroom (FC) in comparison to traditional lectures (TL). Eight obstetrics and gynecology lectures were selected for the flipped classroom. Half of the topics were assessed using MCQs, and the other half used the objective structured clinical examination (OSCE). The overall mean score of FC was better than for TL (47.3+/-6.1 vs. 42.7+/-5.9; p<0.0001). Sixty percent of the participants showed increased satisfaction with the FC [30].

A modified cross-over study was done among third-year medical undergraduates to analyze the effectiveness of TBL over passive learning in gaining knowledge on neurological localization and emergencies. Out of 49 total students, 55.1% were males, and the mean age was 21.4 years. Mean percentage change in score from baseline pre-test was significantly better in the TBL for both post-tests, taken immediately (p=0.023) and after 48 hours (p=0.001). Another interesting finding was a significant increase in post-test scores after the TBL sessions in a group of weaker students [38].

Emergency medicine residents from post-graduate years one to four were randomized into two groups. A cross-over study was performed with a 50-minute powerpoint-based lecture and flipped classroom module (20-minute at-home video and 30-minute case-based discussion). Modules were based on low back pain and headache. The low back pain module did not show a significant difference in scores compared to the headache module. Hence, the result was contradictory [20].

In the United States, third-year medical students rotating in pediatrics were checked for the effect of TBL in improving scores on exams in comparison to traditional didactic lectures for the blood disorders module. Institutional TBL score was significantly better as compared to the national score (0.70+/-0.21 and 0.64+/-0.15), respectively, with a p-value of 0.031 [41].

Medical students from the Medicine School of Chifeng College were divided into TBL (N=98) and TL (N=99) groups for anatomy learning. The male to female population was almost equally distributed in both groups. The average scores out of 100 in the TBL and TL groups were 81.70+/-8.53 and 74.4+/-8.27, respectively, at the statistically significant level of p<0.01. The study also fostered that the TBL session enhanced communication among peers and teachers [14].

A total of 111 third-year medical undergraduates in China were divided into TBL (N=55) and lecture-based learning (LBL) (N=56). Two individual terminal tests (ITT I and ITT II) were taken immediately after the class and the other one week after the class. ITT I did not show a significant difference, but ITT II showed a significant difference in TBL vs. LBL (19.15+/-3.93 vs. 17.46+/-4.65; p=0.042). A survey after TBL completion showed that a good percentage of students had increased interest in learning, ability to solve problems, and effective communication skills [15].

A cross-sectional study in dental students to determine if clinical reasoning and fact-based knowledge questions used in TBL augment their performance in esthetic dentistry consisted of 52 women and 45 men. They were randomly assigned to 18 groups with five or six students each in a group. Seven TBL sessions were organized in four steps, and the outcome was measured in comparison to higher and lower GPAs of students. The correct answer rate in the final exam, IRAT, and TRAT were more elevated in high GPA students than low GPA students. Still, the degree of improvement of correct answer rate in both groups yielded similar findings [43].

In another study done in India, hundred first-year medical students were randomly stratified into two groups to compare the effectiveness of team-based learning compared to traditional lecture-based education in problem-solving skills, student's perception, and gender influence on the learning method. The "organ function test" was tested with eight short answers problem-solving exercises, after both team-based and lecture-based learning. Scores in tests assessing problem-solving skills were higher in TBL in both high achievers and low achievers groups of students (p<0.05), however scores compared after TBL and traditional lecture without stratification did not show a significant difference. Mean differences were analyzed using the two-sample t-test. More than 70% of students perceived that the TBL session was interesting, encouraging, motivating, stress-free, and effective in this study. However, less than 50% of students perceived that TBL should replace all lecture sessions [16].

A study was done by Brandler et al. to analyze the effectiveness of a team-based learning approach among pathology residents. A total of four, two hours TBL sessions were held, preceded by self-learning of the material and learning objectives of the session. IRAT and GRAT were compared using Wilcoxon matched-pairs signed-rank tests for the first through fourth TBL sessions. Residents scored comparatively higher when they were learning in teams [31].

Vázquez-García et al. performed a study to incorporate collaborative-teaching activity into multiple systems in the classroom. Sixty-nine second-year medical students attended both regular lecture-based classes and collaborative-group learning. Multiple-choice tests after each subtopic were taken in the individual phase (IP) and the collaborative phase (CP) differently wherein CP, students were allowed to team up to come up with quiz answers. The average score observed using t-test in CP was found to be 70% greater than in IP (average subtopic quizzes score CP vs. IP 69.8+/-2.7 vs. 47.2+/-2.2, P < 0.001 and average global assessment quizzes score CP vs. IP 61.0+/-0.6 vs. 44.8+/-0.8, p<0.001). Data also showed that the collaborative approach to teaching was effective in retention and understanding the concept [32].

Chandelkar et al. included undergraduate bachelor in dental surgery (BDS) students to study the effectiveness of small group teaching in pharmacology and promote its implementation for a better academic experience. The study population was a small group of 15 students. The usual didactic lecture was followed by the test (test I), self-directed learning was followed by the same test (test II), and, lastly, a small group teaching was followed by the same test (test III). Mean percentage scores of tests I, II, and III were 27.83, 50.66, and 78.66, respectively, out of forty MCQs. A substantial percentage of people thought that small group teaching helped answer the MCQ tests, improved learning, and showed interest in similar exercises in the future [33].

In Denmark, a study was done to analyze and compare the impact of quiz-based and conventional teaching methods in a laboratory exercise. A total of 155 second-year medical students volunteered; 34% were males, and 66 % were females. They were divided into three groups: students doing individual quizzes (N=57), students doing group quizzes (N=56), and controls (N=42). The study revealed that students doing individual quizzes performed better than those doing group quizzes; however, students' satisfaction was higher during group quizzes. Kruskal-Wallis test was used to analyze the differences between the groups [34].

A comparison was made in a study conducted in Australia between team-based learning and problem-based learning to test team collaboration. Twenty first-year medical students participated in the study. PBL session was a four-week program conducted in a traditional format, and TBL was a two-week session with four teams comprising five students each. Twenty students participated in the study with a follow-up rate of 95%. The use of small groups, the readiness assurance tests, immediate feedback from an expert clinician, and time efficiency were all aspects of the TBL experience that students found positive. There was an improvement in test scores through the application of team-based learning [8].

Another study in the United States compared team-based learning with the mixed active learning (MAL) method for ambulatory care. Sixty-four students participated in the survey, with 37 in TBL and 27 in diverse, dynamic learning formats (journal club presentation, group/class discussion). TBL was a twice-weekly three-hour session, and MAL was a thrice-weekly two-hour session. No significant difference in cumulative GPA was noted among the two groups (TBL-3.30 vs. MAL 3.14; p=0.83). Students' performances were compared based on their grade points adjusted for confounding by their prior exam scores. TBL group was assessed based on attendance, IRAT, and TRAT, and mixed active learning group was evaluated based on exam scores. TBL group earned 0.33 more quality points than the MAL group [18].

A modified team-based approach was incorporated in training fellows of pediatrics endocrine fellowship. The fellows were divided into two teams with equitable distribution of years of training in each group. A significant difference was noted on IRAT/IAPP mean scores by years of training (p<0.05) [17].

An evaluation of the live versus virtual team learning approach was performed in Singapore. A total of 60 participants comprising of equally medicine and nursing students participated. The mean age was 22.17±2.07 (live group: 21.82±1.07, virtual group: 22.53±2.70; p= 0.06). Eighty-one (67.5%) were female. Third and fourth-year students' distribution was homogenous in both groups. The demographic variation between the two groups was not statistically significant (except age). A paired sample t-test was applied to examine significant changes between the baseline and post-test performance scores and an independent sample t-test to determine differences in the post-test scores between the groups. The team-based simulation assessment revealed no significant differences between the virtual and simulation groups' communication performance post-test scores (p=0.29). There were significant increases in inter-professional attitudes post-test scores from the baseline scores in both groups, with no significant differences over the three-time points [19].

An integrated learning approach combining team-based learning with case-based learning was studied in Iran among nursing students studying psychiatry. It comprised 26 females out of 41 participants of the age group 20-25 years. There was an increase in the students' self-directed learning based on their performance on the post-test. The results showed that the students' self-directed learning increased after the intervention. The mean difference before and after intervention self-management was statistically significant (p=0.0001). Also, self-regulated learning increased with the mean difference after intervention (p=0.001) [2].

Third-year medical students studying psychiatry were evaluated for team-based learning in the United States. Males represented 64.93% of participants among 20 total students. Eight of 16 regular traditional learning methods were replaced with team-based learning and the five cohorts. Each cohort rotated every fifth week for one week in one of two ambulatory clinics, including a patient-centered medical home and a hospital-based clinic. Scores were compared using ANOVA with a post hoc Duncan's multiple comparison test. Implementation of team-based learning helped in higher scores in the National Board of Medical Examiners Psychiatry test, and students perceived team learning activities to be more effective and enjoyable [42].

Similarly, Baylor College of Medicine conducted a case-control study to compare team learning and lecture-based learning among medical students and physician assistants. Eight sessions, each lasting 50-120 minutes for lecture, and nine sessions each lasting 50-120 minutes for team learning were conducted. The behavior pattern was uniform across first/second-year medical students and physician assistants. The amount of learner-to-learner engagement in PBL and team learning was similar and much more significant than in lectures, where most meetings were of the learner-to-instructor and self-engagement types. Also, learner-to-instructor engagement appeared greater in team learning than in PBL [3].

A case-control study was conducted in the United States comparing individual professional outcomes to collaborative outcomes if medicine and nursing students worked together. A 20-minute teaching session on physician and nursing team learning approach, team interaction and patient-focused care, team communication, collaborative skill performance including barriers to successful medical teamwork. In addition, video examples of different types of team interaction on resuscitation cases on cardiac arrest and ICU resuscitation were made available. Debriefing sessions followed this up. Outcomes were computed, and the means of the two approaches were compared. The collaborative team achieved significant improvement in critical actions gained 6.5 of the eight critical actions in a mean time of 19.4 minutes [37].

Discussion

Our systematic review evaluated the impact of team-based learning among various health care professionals, including medical students, fellows, residents, nurses, dentists, students, attending physicians, etc., regarding knowledge scores and learners' attitudes towards team-based learning. We found that more than two-thirds of the included studies reported improved academic performance in terms of scores among those enrolled in team-based learning compared to traditional lecture-based knowledge. This finding was consistent with different disciplines of medicine, including neurology, psychiatry, anatomy, pathology, pediatrics. It was also consistent among the participants with various education levels, including undergraduate medical students, medical residents, fellows, and attending physicians. Our findings of improved academic performance, skills, and knowledge scores were concordant with previous systematic reviews and meta-analyses done by Alberti et al., Chen et al., and Fatmi et al. [9,46,47]. Only two studies showed contradictory findings. Riddell et al. showed no difference in score performance among emergency medicine residents who underwent it traditionally. They flipped the classroom module, and Berg et al. found that medical students performed better in individual quizzes than group quizzes [20,34]. Newer modalities of team-based learning using online module was similar to traditional in-person learning. At the same time, there was no difference in score performance among nursing students between learning with live simulation and virtual module in nursing students [12,19]. Team-based learning has excellent application in medical education because it facilitates learning with a higher teacher-to-student ratio without constraining the health resources. Team-based learning is also being used in clinical practice. Milzman et al. found that collaborative learning with medicine and nursing students led to significant improvement in critical actions leading to better patient care [37].

Another crucial facet of team-based learning is the learner's attitude towards such learning modality compared to traditional learning methods. About twenty studies reported favorable responses of medical professionals towards team-based learning. Multiple studies included in our review highlighted that team-based learning improved the engagement, understanding, and communication skills among health care professionals and medical students. Medical students, residents, nurses, dental students, and physicians gave positive feedback regarding improved interest, motivation, self-directed learning, time efficiency, and greater time allocation to teaching and learning activities when they participated in team-based learning activities. Similar findings of improved communication and self-directed learning were seen in the review done by Alberti et al. [47]. However, Fatmi et al. reported no certain benefit in learner reaction with team-based learning [46]. Constraints can explain this with the inclusion criteria of Fatmi et al., restricting the inclusion to the validated definition of team-based learning alone [46]. Team-based learning enhanced problem-solving skills among medical students as per Jeng et al. [15]. Four studies reported improvement in individual reassurance test and group reassurance test with team-based learning compared to traditional-based learning [13,22,26,27].

Our systematic review is comprehensive because it included many studies with various designs and encompassed a wide range of health care professionals in several countries. However, there were a few limitations. The heterogeneity in our study is explained by various study designs, study populations, and different modalities of team-based learning. Most of the included studies had a low sample size and were non-randomized. In addition, there were limitations like lack of control group and generalizability.

## Conclusions

Team-based learning is instrumental in medical education, enhancing academic performance, communication skills, and clinical outcomes. It also strengthens learner engagement, motivation, and satisfaction as compared to traditional lecture-based learning.

## Appendices

Appendix 1: electronic search details

Embase

Search: ('team learning' OR 'collaborative learning'/exp OR 'collaborative learning' OR 'cooperative knowledge') AND ('health care workers' OR 'health care professionals' OR 'medicine'/exp OR 'medicine')

Hits: 1155

Link: https://www.embase.com/?org.apache.catalina.filters.CSRF_NONCE=8C91A8CA30D6F27C5D756D90BBB72055#advancedSearch/resultspage/history.2/page.1/25.items/orderby.date/source.

Scopus

Search: ("team learning" or "collaborative learning" OR "cooperative knowledge") AND ("health care workers" OR "health care professionals" OR "Medicine")

Hits: 370

Link:

https://www.scopus.com/results/results.uri?src=s&st1=&st2=&sot=b&sdt=b&origin=searchbasic&rr=&sl=161&s=TITLE-ABS-KEY%20((%22team%20learning%22%20or%20%22collaborative%20learning%22%20OR%20%22cooperative%20knowledge%22)%20AND%20(%22health%20care%20workers%22%20OR%20%22health%20care%20professionals%22%20OR%20%22Medicine%22))

Pubmed

Search: ("team learning" or "collaborative learning" OR "cooperative knowledge") AND ("health care workers" OR "health care professionals" OR "Medicine")

Hits: 410

Link: https://pubmed.ncbi.nlm.nih.gov/?term=%28%22team+learning%22+or+%22collaborative+learning%22+OR+%22cooperative+knowledge%22%29+AND+%28%22health+care+workers%22+OR+%22health+care+professionals%22+OR+%22Medicine%22%29

Pubmed Central

Search: ("team learning" or "collaborative learning" OR "cooperative knowledge") AND ("health care workers" OR "health care professionals" OR "Medicine")

Details: ("team learning"[All Fields] OR "collaborative learning"[All Fields] OR "cooperative knowledge"[All Fields]) AND ("health care workers"[All Fields] OR "health care professionals"[All Fields] OR "Medicine"[All Fields])

Hits: 2226

Link: https://www.ncbi.nlm.nih.gov/pmc/?term=(%22team+learning%22+or+%22collaborative+learning%22+OR+%22cooperative+knowledge%22)+AND+(%22health+care+workers%22+OR+%22health+care+professionals%22+OR+%22Medicine%22)
